# PARN Maintains RNA Stability to Regulate Insulin Maturation and GSIS in Pancreatic β Cells

**DOI:** 10.1002/advs.202407774

**Published:** 2024-09-19

**Authors:** Xiaomei Xie, Xuexue Chen, Chaofan Wang, Longjie Sun, Weiru Yu, Zheng Lv, Shuang Tian, Xiaohong Yao, Fengchao Wang, Deqiang Ding, Juan Chen, Jiali Liu

**Affiliations:** ^1^ State Key Laboratory of Animal Biotech Breeding College of Biological Sciences China Agricultural University Beijing 100193 China; ^2^ College of Food and Bioengineering Fujian Polytechnic Normal University Longjiang Street Fuqing Fujian 310300 China; ^3^ Key Laboratory of Precision Nutrition and Food Quality Department of Nutrition and Health China Agricultural University Beijing 100190 China; ^4^ National Institute of Biological Sciences Beijing 102206 China; ^5^ Tsinghua Institute of Multidisciplinary Biomedical Research Tsinghua University Beijing 102206 China; ^6^ Shanghai Key Laboratory of Maternal Fetal Medicine Clinical and Translational Research Center Shanghai First Maternity and Infant Hospital Frontier Science Center for Stem Cell Research School of Life Sciences and Technology Tongji University Shanghai 200092 China

**Keywords:** β cell, insulin maturation and secretion, RNA binding protein

## Abstract

Diabetes, a metabolic disorder characterized by hyperglycemia, underscores the importance of normal pancreatic β‐cell development and function in maintaining glucose homeostasis. Poly(A)‐specific ribonuclease (PARN) serves as the principal regulator of messenger RNA (mRNA) stability, yet its specific role in pancreatic β cells remains unclear. This study utilizes mice with targeted PARN deficiency in β cells to elucidate this role. Notably, *Parn* conditional knockout mice present unaltered β‐cell development and insulin sensitivity but reduced glucose‐stimulated insulin secretion (GSIS). The observed outcomes are corroborated in NIT‐1 cells. Furthermore, transcriptomic analyses reveal aberrant mRNA expression of genes crucial for insulin secretion in PARN‐deficient β cells. Insights from linear amplification of complementary DNA ends and sequencing and coimmunoprecipitation experiments reveal an interaction between PARN and polypyrimidine tract‐binding protein 1 (PTBP1), regulating the RNA stability of *solute carrier family 30, member 8* (*Slc30a8*) and *carbohydrate sulfotransferase 3* (*Chst3*). Interference with either PARN or PTBP1 disrupts this stability. These data indicate that PARN deficiency hampers GSIS and insulin maturation by destabilizing *Slc30a8* and *Chst3* RNAs. These findings provide compelling evidence indicating that PARN is a potential therapeutic target for enhancing insulin maturation and secretion.

## Graphical abstract

This study elucidates that poly(A)‐specific ribonuclease (PARN) interacts with polypyrimidine tract‐binding protein 1 to directly bind and maintain the messenger RNA stability of *solute carrier family 30, member 8* and *carbohydrate sulfotransferase 3*in β cells, thereby regulating insulin maturation and secretion. It is suggested that PARN is a potential therapeutic target for diabetes.

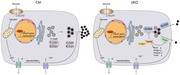

## 1 Introduction

Diabetes manifests as a multifaceted metabolic disorder characterized by chronic hyperglycemia and is typically categorized into gestational diabetes, CNOT^[^ ^1^ ^]^ type 1 diabetes mellitus,^[^ ^2^, ^3^ ^]^ type 2 diabetes mellitus (T2DM),^[^ ^4^ ^]^ or other specific types.^[^ ^5^ ^]^ The etiology involves disruptions in insulin secretion, abnormal insulin activity, or a combination of both factors.^[^ ^6^ ^]^ Mature insulin, comprising a 30‐residue B chain and a 21‐residue A chain interconnected by two disulfide bonds, plays a pivotal role in glucose regulation.^[^ ^7^ ^]^ Islets constitute ≈1–2% of the pancreatic volume, and house β cells, which account for 60–70% of the islet cell population.^[^ ^8^ ^]^ The synthesis and secretion of insulin by β cells are essential for sustaining normal metabolic processes in organisms.

The synthesis of insulin in response to glucose involves intricate regulation at multiple levels, including transcriptional, translational, and posttranslational processes.^[^ ^9^ ^]^ RNA‐binding proteins (RBPs), a diverse group of proteins with various structures and functions, play crucial roles in the posttranscriptional control of both mRNAs and noncoding RNAs. Their influence extends to RNA splicing, transport, modification, stability, and translation.^[^ ^10^ ^]^ Notably, among posttranscriptional regulators are RBPs such as ELAV like RNA binding protein 4 (HuD), protein disulfide isomerase (PDI), and polypyrimidine tract‐binding protein 1 (PTBP1), which were identified for their roles in insulin expression by binding to the untranslated region (UTR) of insulin mRNA.^[^ ^11^ ^]^ The *cis*‐acting sequence element in the mRNA UTR collaborates with *trans*‐acting factors, including microRNAs and RBPs, to dynamically modulate the translation of β cells in response to external stimuli, thereby contributing to glucose homeostasis. Key regulators such as CUG‐binding protein 1 activate phosphodiesterase 3B (PDE3B), influencing insulin secretion,^[^ ^12^ ^]^ whereas DEAD box 1 (DDX1)‐mediated inhibition of insulin translation exacerbates insulin resistance in individuals with obesity, leading to elevated blood glucose levels.^[^ ^13^ ^]^ These findings underscore the essential role of RBPs in maintaining the functionality of mouse β cells.

Within eukaryotic cells, the equilibrium between mRNA synthesis and degradation is pivotal for mRNA homeostasis. Deadenylation, the removal of adenosine residues from the poly(A) tail, represents the primary and critical step in the mRNA degradation pathway.^[^ ^14^ ^]^ In mammals, the carbon catabolite repression 4 (CCR4)‐negative on TATA‐less (NOT) complex serves as the principal deadenylase, with CNOT1 being a key catalytic subunit.^[^ ^15^ ^]^ Studies have indicated that mice deficient in CNOT1 exhibit diminished quality white adipose tissue and brown adipose tissue, underscoring the role of the CCR4‐NOT complex in regulating gene expression and the subsequent impacts on insulin sensitivity, lipid metabolism, and body temperature control.^[^ ^16^ ^]^ However, the influence of deadenylases on insulin synthesis and secretion remains unclear. Poly(A)‐specific ribonuclease (PARN), a prominent mammalian deadenylase, uniquely binds both the 5′ cap structure and 3′ poly(A) tail, increasing the mRNA degradation rate and processability.^[^ ^17^ ^]^ Consequently, our investigation sought to elucidate whether PARN plays a discernible role in the processes of insulin synthesis or secretion.

In our current investigation, we initially observed a reduction in insulin secretion in *Parn*‐knockdown NIT‐1 cells subjected to high‐glucose conditions. We subsequently generated mice with β‐cell‐specific *Parn* knockout to systematically assess the role of PARN in β‐cell function. Intriguingly, the absence of PARN did not yield discernible alterations in islet morphology, β‐cell mass, islet size, or insulin sensitivity in the murine model. However, it manifested as glucose intolerance, insulin immaturity, and a noteworthy reduction in glucose‐stimulated insulin secretion (GSIS). These findings underscore the specific impact of PARN deficiency on aspects of glucose metabolism and insulin secretion without overtly affecting key structural and sensitivity parameters in the β cells of mice.

## 2 Results

### 2.1 PARN Inhibition Impairs Glucose‐Stimulated Insulin Secretion in NIT‐1 Cells

**Figure 1:**
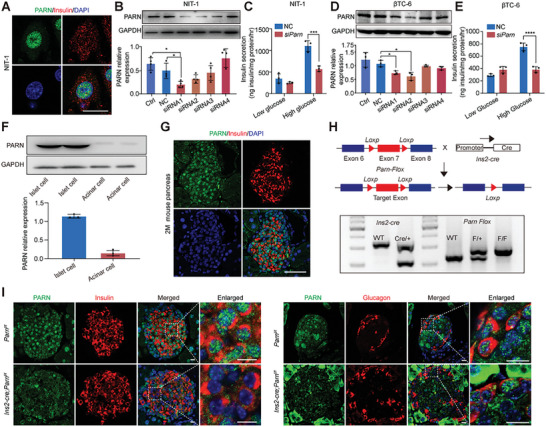
PARN inhibition impairs glucose‐stimulated insulin secretion in NIT‐1 and β‐TC6 cells and PARN was successfully selectively deleted in β cell. A) Immunofluorescence double staining NIT‐1 cells with antibodies against insulin (red), PARN (green), nuclei were stained with 4',6‐diamidino‐2‐phenylindole (DAPI) (blue). B) Western blotting analysis was performed to detect the expression level of PARN protein in NIT‐cells transfected with siRNA 48 h, *n* = 3–4. C) Insulin ELISA kit was used to detect the insulin secretion of NIT‐cells transfected with *Parn* siRNA1, *n=3*. D) Western blotting analysis was performed to detect the expression level of PARN protein in β‐TC6 cells transfected with siRNA 48 h, *n* = 3. E) Insulin ELISA kit was used to detect the insulin secretion of β‐TC6 cells transfected with *Parn* siRNA2, *n* = 3. F) Western blotting was used to analyze protein expression in islets and acinar cells, quantitative data from three independent experiments. G) 2 months pancreatic sections were subjected to co‐IF staining using antibodies against insulin (red), PARN (green), nuclei were stained with DAPI (blue). H) Knockout scheme design in mice and polymerase chain reaction, the DNA templates came from mice tails. I) Co‐immunofluorescent images of pancreatic sectioned islets from 18 weeks old Ctrl and cKO mice showing PARN (green), insulin (red), glucagon (red), and nuclei were stained with DAPI (blue), bar = 25 µm. ns *p* ≥ 0.05, *p < 0.05, ***p < 0.001, ****p < 0.0001, Student's *t*‐test and ANOVA.

We initially examined the expression of PARN in NIT‐1 cells through immunofluorescence (IF) to assess the role of PARN in β cells, and the results revealed that PARN was expressed mainly in the nucleus (**Figure** **1**A). We subsequently confirmed a substantial reduction in *Parn* mRNA (Figure S1A, Supporting Information) and protein expression levels upon the transfection of *Parn* small interfering RNAs (siRNAs) into NIT‐1 cells, with siRNA1 generating the most pronounced effect on gene expression (Figure 1B). Notably, there was no difference in insulin secretion between the PARN‐deficient NIT‐1 cells and the negative control (NC) cells in the presence of low glucose, but after 1 h in the presence of high glucose, insulin secretion was significantly reduced. When the background under low‐glucose conditions was subtracted from the high‐glucose samples, insulin secretion was decreased by ≈75% compared with that of the NC group (Figure 1C). Additionally, we performed insulin secretion experiments with β‐TC6 cells, and the results revealed that *Parn* knockdown in β‐TC6 cells did not affect basal insulin secretion, but GSIS decreased (Figure 1D,E), which was consistent with the results in NIT‐1 cells. These findings underscored the functional involvement of PARN in the process of GSIS.

### 2.2 PARN Was Specifically Knocked Out in Mouse β Cells

We explored the functional role of PARN in mouse β cells by evaluating its expression across various mouse tissues through Western blotting. The results revealed increased expression of PARN in the mouse pancreas, with particularly elevated protein levels observed in the islets (Figure S1B (Supporting Information) and Figure 1F). Further characterization of PARN expression patterns involved IF costaining of PARN and insulin, which revealed robust expression of PARN in β cells at different time points (Figure S1C (Supporting Information) and Figure 1G). These findings suggest a potential role for PARN in β cells.

We generated mice with specific *Parn* knockout in β cells by establishing insulin 2 (*Ins2)‐cre; Parn^f/f^* mice through crosses of loxp‐flanked *Parn ^f/f^* mice with *Ins2‐cre* mice. These mice were designated *Parn*‐specific knockout mice in β cells (cKO), whereas *Parn ^f/f^* mice and other littermates served as controls (Ctrl) (Figure 1H). The negative IF costaining of PARN and insulin and the positive one with glucagon (Figure 1l). These results indicated the successful construction of a mouse model featuring specific *Parn* knockout in β cells.

### 2.3 Specific Knockout of *Parn* in β Cells Is Associated with Glucose Intolerance but Not with Insulin Resistance

**Figure 2:**
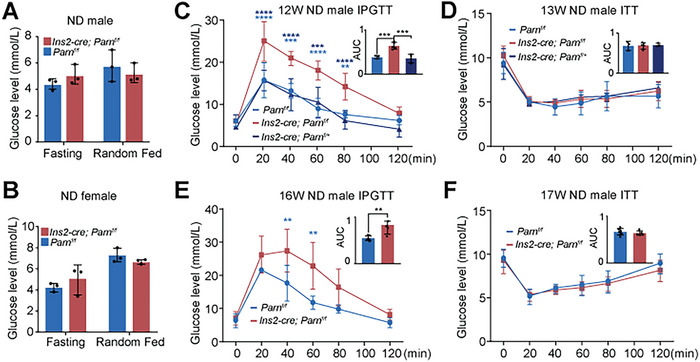
β‐cell specific knockout *Parn* induces glucose intolerance but not insulin sensibility. A) Blood glucose concentrations in fasting and free‐feeding ND male Ctrl and cKO mice, *n* = 3. B) Blood glucose concentrations in fasting and free‐feeding ND female Ctrl and cKO mice under normal feeding conditions, *n* = 3. C,E) Blood glucose excursions during a 2 g kg^−1^ glucose tolerance test of (C) 12, (E) 16 weeks male mice and the area under the curve (AUC), *n* = 3–5. D,F) Insulin tolerance test of (D) 13, (F) 17 weeks male mice and the AUC, *n* = 3–6. **p < 0.01, *** *p* < 0.001, ****p < 0.0001, Student's *t*‐test and ANOVA.

To evaluate the impact of β‐cell‐specific *Parn* deletion in mice, we assessed blood glucose levels in mice fed with a normal diet (ND). Initially, fasting and ad libitum blood glucose levels were comparable between the cKO and Ctrl mice (**Figure** **2**A,B). However, glucose tolerance was significantly impaired in the cKO mice at 12 weeks of age, in *Ins2‐cre; Parn ^f/+^* mice, glucose levels did not differ from those in *Parn ^f/f^* mice (Figure 2C). This finding is consistent with previous reports indicating glucose intolerance in *Ins2‐cre* mice.^[^ ^18^ ^]^ Similarly, at 16 weeks of age, cKO mice presented significantly higher blood glucose concentrations than Ctrl mice (Figure 2E). Importantly, the loss of PARN in β cells did not influence insulin sensitivity, as evidenced by the insulin tolerance test (ITTs) conducted at 13 and 17 weeks of age (Figure 2D,F). Similar findings were observed in female *Parn* cKO mice (Figure S2, Supporting Information). These results illustrated that β‐cell‐specific *Parn* knockout induced glucose intolerance without affecting insulin sensitivity in ND‐fed mice.

### 2.4 PARN Is Not Required for β‐Cell Development

**Figure 3:**
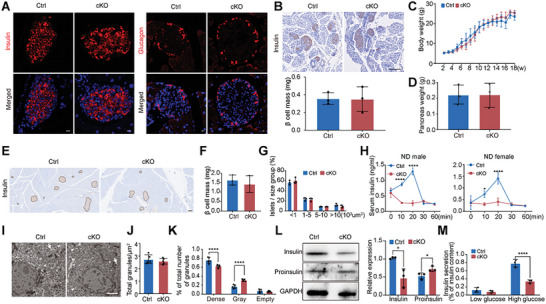
Selective deletion of *Parn* in pancreatic β cells leads to impaired glucose‐stimulated insulin secretion. A) Immunofluorescent images of pancreatic sectioned from 18 weeks Ctrl and cKO mice showing insulin (red, left), glucagon (red, right), and nuclei (DAPI, blue), bar = 10 µm. B) Representative images of IHC pancreatic sections and the statistical results of β‐cell mass at P3 Ctrl and cKO mice, bar = 100 µm, *n* = 3. C) Body weight from 2 to 18 weeks Ctrl and cKO mice, *n* = 4. D) Pancreas weight at 18 weeks Ctrl and cKO mice, *n* = 3. E) IHC of insulin pancreatic sections from 18 weeks old Ctrl and cKO mice, bar = 100 µm. F) β‐cell mass was calculated in 18 weeks Ctrl and cKO mice, *n* = 3. G) Islet size distribution as stratified by ranges of islet area from 18 weeks Ctrl and cKO mice by ImageJ, *n* = 3. H) Serum insulin level at fasting, 10, 20, 30, and 60 min after glucose injection from 18 weeks old male and female Ctrl and cKO mice, *n* = 3–4. I) The transmission electron microscopy images are from 18 weeks old Ctrl and cKO mice, with arrows indicating mature insulin secretion granules, arrow heads indicating immature insulin secretion granules, arrows indicating empty granules, bar = 2 µm. J) The number of total insulin granules calculated from the vesicle area, *n* = 5. K) The percentage of dense‐core, gray‐core, and empty insulin granules calculated from the vesicle area, *n* = 5. L) The insulin and proinsulin protein level of cKO and Ctrl mice was detected by Western blotting, *n* = 3. M) Insulin secretion assay on islet from 18 weeks old Ctrl and cKO male mice with 3 and 20 mm glucose in vitro. Insulin secretion/intracellular insulin content ratio, bar = 20 µm, *n* = 3. *p < 0.05, ****p < 0.0001, Student's *t*‐test and ANOVA.

We subsequently explored whether the observed impaired glucose tolerance in cKO mice stemmed from alterations in β‐cell mass and islet size. IF staining of pancreatic sections from cKO and Ctrl mice for insulin and glucagon revealed that *Parn* deletion did not influence the distribution of α or β cells within the islets (**Figure** **3**A). At 3 days, the β‐cell mass was not significantly different between the cKO and Ctrl mice (Figure 3B). Comparable body weights (Figure 3C) and pancreatic weights (Figure 3D) were compared between the two groups at 18 weeks. Furthermore, at 18 weeks, pancreatic sections subjected to insulin immunohistochemistry (IHC) staining displayed no significant differences in β‐cell mass (Figure 3E,F) or the distribution of islet size (Figure 3G) between cKO and Ctrl mice. A parallel phenotype was observed in female mice (Figure S3A–F, Supporting Information). These findings suggested that *Parn*‐specific deletion in β cells had no discernible effect on the morphology or development of islets.

### 2.5 Loss of PARN in β Cells Causes GSIS Deficiency

We investigated insulin secretion through a GSIS assay to elucidate the mechanism underlying impaired glucose tolerance in cKO mice with β‐cell *Parn* deficiency. Enzyme‐linked immunosorbent assays (ELISAs) revealed no significant difference in the serum insulin concentration between the Ctrl and cKO mice after fasting, but both the female and male cKO mice presented reduced serum insulin levels in response to glucose in vivo (Figure 3H). The intracellular islet insulin concentration was assessed via transmission electron microscopy (TEM) (Figure 3I) and quantified by analyzing insulin granules with ImageJ. The total number of insulin‐secreting granules in the cKO mice did not decrease (Figure 3J), but a decrease in the number of mature insulin‐secreting granules accompanied by an increase in the number of immature insulin‐secreting granules was observed (Figure 3K). The islets of cKO and Ctrl mice were isolated to analyze the expression levels of insulin and proinsulin. The level of insulin protein in the islets of cKO mice significantly decreased, whereas the expression of proinsulin protein increased significantly (Figure 3L). These results were consistent with our TEM observations, providing further evidence for the presence of insulin maturation disorders in cKO mice.

Additionally, islet cells that had been isolated and cultured in vitro presented a decreased ratio of secreted insulin to intracellular insulin in the presence of 20 mm glucose (Figure S3G (Supporting Information) and Figure 3M). These findings suggested that the absence of PARN in β cells resulted in diminished GSIS and impaired insulin maturation.

### 2.6 Dysfunction of GSIS in High‐Fat‐Diet (HFD)‐Fed cKO Mice

**Figure 4:**
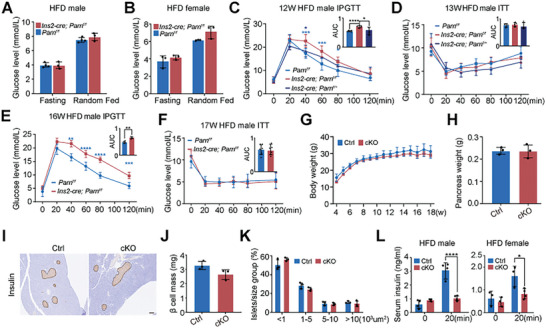
Impairment of glucose‐induced insulin secretion in β‐cell‐specific *Parn*‐knockout mice in high fat diet. A,B) Blood glucose level in fasting and free‐feeding male (A) mice and female (B) HFD Ctrl and cKO mice, *n* = 3–4. C,E) Blood glucose levels during IPGTT in 12 weeks (C) and 16 weeks (E) Ctrl and cKO mice, with calculated AUCs, *n* = 4–6. D,F) Blood glucose levels during ITT in 13 weeks (D) and 17 weeks (F) Ctrl and cKO mice and the AUC, *n* = 4–6. G) Body weight of Ctrl and cKO mice during 15 weeks of HFD, *n* = 3–6. H) Pancreas weight at 18 weeks of age, *n* = 3. I) IHC pancreatic sections from 18 weeks Ctrl and cKO mice, bar = 100 µm. J) β‐cell mass in entire pancreas from 18 weeks Ctrl and cKO mice, *n* = 3. K) Islet size distribution as stratified by ranges of islet area from 18 weeks Ctrl and cKO mice by ImageJ, *n* = 3. L) Serum insulin was measured in 18 weeks old Ctrl and cKO mice at fasting and 20 min after glucose injection, *n* = 3–4. *p < 0.05, **p < 0.01, ***p < 0.001, ****p < 0.0001, Student's *t*‐test and ANOVA.

Obesity, a critical risk factor for diabetes, contributes to metabolic disorders.^[^ ^19^ ^]^ We fed both Ctrl and cKO mice a HFD from 4 to 18 weeks of age to investigate the impact of a metabolic challenge on β‐cell *Parn* deficiency. The fed and fasting blood glucose levels were similar between the Ctrl and cKO mice (**Figure** **4**A,B). Nevertheless, at 12 and 16 weeks of age, cKO mice displayed glucose intolerance (Figure 4C,E). ITTs revealed no discernible differences in blood glucose levels between cKO and Ctrl mice (Figure 4D,F), indicating that impaired systemic glucose homeostasis in cKO mice was unrelated to insulin sensitivity. We compared body weights between cKO and Ctrl mice to explore the potential causes of glucose intolerance and found no significant differences (Figure 4G). There were no significant differences in pancreatic weight, β‐cell mass, or islet size distribution, as assessed by insulin staining of pancreatic sections, between the cKO and Ctrl mice (Figure 4H–K). Serum insulin measurements further supported these findings, with cKO mice showing consistent fasting insulin levels but significantly lower insulin levels than Ctrl mice following glucose stimulation (Figure 4L). Notably, these observations were also observed in female mice (Figure S4, Supporting Information). Collectively, these results suggested that HFD‐fed cKO mice maintained normal β‐cell development and that their impaired glucose homeostasis in response to glucose stimulation was attributed primarily to a substantial reduction in insulin secretion. Importantly, the cKO mouse phenotype was not exacerbated by obesity.

### 2.7 Loss of PARN in β Cells Results in Decreased Expression Levels of Genes That Affect Insulin Secretion

**Figure 5:**
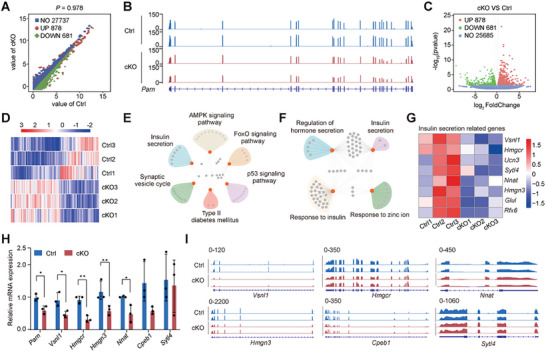
Loss of PARN in β cells results in decreased expression levels of genes that affect insulin secretion. A) The scatter plot shows the RNA‐seq Pearson correlation. B) Integrative Genomics Viewer (IGV) visualizes *Parn* reads values in cKO and Ctrl groups. C) Volcanic map showing the expression of the 878 genes up and 681 genes downregulated in cKO islet. D) Heat map showing differentially expressed genes in cKO islet at 12 weeks old. E) List of KEGG pathway enrichment analysis. F) Selected molecular functions enriched in different (DE) genes, identified by Gene Ontology analysis. G) Heat map of selected DE genes related to insulin secretion. H) The mRNA expression level of insulin‐secretion‐related genes was verified by qRT‐PCR, *n* = 3. I) Representative IGV browser tracks of gene peaks. For each panel, blue tracks are from Ctrl mice, red tracks are from cKO mice. *p < 0.05, **p < 0.01, Student's *t*‐test and ANOVA.

We conducted RNA‐seq profiling of islets from Ctrl and cKO mice to elucidate the underlying mechanisms by which PARN influences insulin secretion. An analysis of differentially expressed genes (DEGs) revealed robust repeatability across all three replicates within the same genotypes (**Figure** **5**A). *Parn* mRNA expression was notably reduced, and the expression of 878 genes, such as synaptoporin and nitric oxide synthase 1, was upregulated, whereas the expression of 681 genes, such as proprotein convertase subtilisin/kexin type 9 and visinin‐like protein‐1 (*Vsnl1*), was downregulated in cKO islets (Figure 5B,C). Hierarchical clustering of DEGs between Ctrl and cKO mice is visually represented in a heat map (Figure 5D). For deeper insights, we performed gene ontology (GO) and Kyoto Encyclopedia of Genes and Genomes (KEGG) enrichment analyses, both of which consistently highlighted insulin secretion (Figure 5E,F). A more granular examination of *Vsnl1*, 3‐hydroxy‐3‐methylglutaryl‐CoA reductase (*Hmgcr*), high‐mobility group N 3 (*Hmgn3*), urocortin 3 (*Ucn3*), synaptotagmin‐like 4 (*Sytl4*), glutamate‐ammonia ligase (*Glul*), regulatory factor X, 6 (*Rfx6*), and neuronatin (*Nnat*), which are related to insulin secretion genes, revealed the expression levels of these genes in each group (Figure 5G). Quantitative real‐time polymerase chain reaction (qRT‐PCR) confirmed the decreased expression of *Vsnl1*, *Hmgcr*, *Hmgn3*, and *Nnat* in the islets of cKO mice (Figure 5H), and their abundance mirrored the observed trends (Figure 5I). These findings indicated that *Parn* deletion disrupted the expression of pivotal genes implicated in insulin secretion.

### 2.8 PARN Interacts with PTBP1 to Regulate Genes Related to Insulin Maturation and Secretion

**Figure 6:**
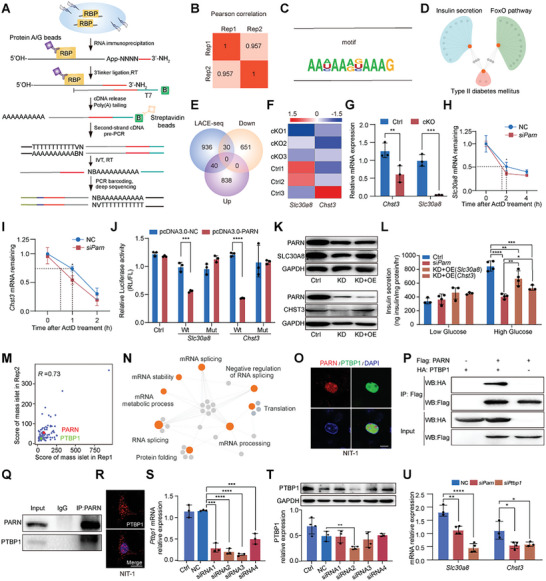
PARN interacting with PTBP1 to regulate the expression of genes during insulin maturation and secretion. A) Schematic of the LACE‐seq method. A circled B represents biotin modification. N, random nucleotide; V can be A, G, or C. B) Pearson correlation between PARN LACE‐seq replicates in total islet for assessing the reproducibility of the data. Pearson correlation for the reads counts of each sample was calculated from two replicates. C) PARN‐binding motifs identified by LACE‐seq in mouse islet. D) Network showing KEGG enrichment analyses of differentially expressed genes. E) Venn diagram showing the overlapped genes between differentially expressed genes and PARN‐binding genes. F) The heat map showed the expression of genes involved in (E) which were related to insulin secretion. G) Relative mRNA expression of *Chst3* and *Slc30a8*, *n* = 3. H) The expression of *Slc30a8* in NIT‐1 cells after transfection with *Parn* siRNA and ActD treatment at different times, *n*  =  3. I) The expression of *Chst3* in NIT‐1 cells after transfection with Parn siRNA and ActD treatment at different times, *n*  =  3. J) The relative signal intensity of Renilla was analyzed in the intracellular luciferase reporting system of HEK293T cells. K) Western blotting analysis was performed to detect the expression level of PARN and *SLC30A8* (*Chst3*) protein in NIT‐1 cells transfected with siRNA and plasmid 48 h. L) Insulin ELISA kit was used to detect the insulin secretion of NIT‐1 cells transfected with *Parn* siRNA2, *n* = 4. M) Pearson correlation plot between PARN co‐IP replicates in islet for assessing the reproducibility of the data. N) GO analysis of the PARN interacting protein corresponding genes. O) PARN–mCherry plasmid and PTBP1–eGFP plasmid were cotransfected in NIT‐1 cells to detect colocalization. P) The interaction of PARN and PTBP1 in 293T cells was detected by Western blotting. Q) Co‐IP detected the endogenous PARN and PTBP1 interaction in NIT‐1 cells. R) Localization of PTBP1 detected by immunofluorescence. S) The knockdown efficiency of NIT‐1 cells transfected with *Ptbp1* siRNA was detected by qRT‐PCR, *n* = 3. T) The knockdown efficiency of NIT‐1 cells transfected with *Ptbp1* siRNA was detected by Western blotting, *n* = 3. U) Expression levels of *Slc30a8* and *Chst3* after transfection with *Parn* or *Ptbp1* siRNA, *n* = 3, bar = 5 µm. *p < 0.05, **p < 0.01, ***p < 0.001, ****p < 0.0001, Student's *t*‐test and ANOVA.

Given the status of PARN as a RBP, we investigated its mechanism of action in islets through linear amplification of complementary DNA ends and sequencing (LACE‐seq)^[^ ^20^ ^]^ (**Figure** **6**A), focusing on islets from 2 months old mice. The two biological replicates within the PARN group exhibited robust correlations with the detected putative binding target RNA molecules, attaining a Pearson coefficient of up to 0.957 (Figure 6B). The most highly enriched motif among these PARN peaks was AA (Figure 6C). A subsequent GO analysis revealed that the PARN‐binding genes were intricately linked to insulin secretion and T2DM (Figure 6D). Integration of the RNA‐seq data with LACE‐seq‐identified peaks revealed 30 downregulated and 40 upregulated transcripts in cKO mice, elucidating direct targets of PARN in islets (Figure 6E and Figure S5A (Supporting Information)). Further scrutiny and validation of these genes revealed direct binding of PARN to solute carrier family 30, member 8 (*Slc30a8*) and carbohydrate sulfotransferase 3 (*Chst3*), both of which were downregulated in cKO mice (Figure 6F,G and Figure S5B (Supporting Information)). The results of the Actinomycin D (ActD) experiment indicated that the half‐lives of *Slc30a8* and *Chst3* mRNAs were shortened after *Parn* knockdown. Depletion of *Parn* in NIT‐1 cells led to a significant increase in the degradation of *Slc30a8* and *Chst3* mRNAs (Figure 6H,I). To demonstrate the direct binding of PARN to *Slc30a8* and *Chst3*, fragments of *Slc30a8* and *Chst3* and their corresponding mutant fragments were amplified and connected to the psi‐Check2 vector. These constructs were then cotransfected with pcDNA3.0‐NC and pcDNA3.0‐PARN into 293T cells. The results of the luciferase assay confirmed the direct binding of PARN to *Slc30a8* and *Chst3* (Figure 6J and Figure S5B (Supporting Information)).

Furthermore, we performed additional experiments in which *Parn* siRNA was transfected alone or in combination with the pcDNA3.0‐*Slc30a8* or pcDNA3.0‐*Chst3* plasmid (Figure 6K). The results showed that *Parn* knockdown led to a decrease in GSIS. Compared with the control, overexpression of *Slc30a8* or *Chst3* also resulted in reduced insulin secretion levels. However, compared with those in the knockdown group, the insulin secretion levels were partially restored (Figure 6L). We knocked down *Slc30a8* or *Chst3* in NIT‐1 cells to further clarify the regulatory mechanism involved, and our cell knockdown model was successfully constructed (Figure S5C,D,F,G, Supporting Information). Moreover, the GSIS results revealed that the insulin level in the supernatant did not differ significantly between the knockdown group and the NC group at low glucose; however, when *Slc30a8* or *Chst3* was knocked down, insulin secretion was significantly lower in the high glucose group than in the control group (Figure S5E,H, Supporting Information).

We conducted immunoprecipitation mass spectrometry to identify proteins influencing insulin secretion that potentially interacted with PARN. The scatterplot revealed strong repeatability between the two sets of experimental data (Figure 6M). GO enrichment analysis of potential interacting proteins highlighted functions related to RNA processing, RNA stability, and RNA splicing (Figure 6N). PTBP1, a crucial RNA‐binding protein that governs mRNA stability and processing, emerged as a noteworthy candidate. The colocalization of mCherry–PARN and green fluorescent protein (GFP)–PTBP1 in NIT‐1 cells was evident upon transfection (Figure 6O). Further experimentation through co‐immunoprecipitation (co‐IP) with FLAG‐PARN and hemagglutinin (HA)–PTBP1 in HEK293T cells confirmed their interaction (Figure 6P). The IP results demonstrated that endogenous PARN interacted with PTBP1 in NIT‐1 cells (Figure 6Q).

We investigated the impact of *Ptbp1* deletion on the mRNA levels of the PARN‐bound targets *Slc30a8* and *Chst3* in NIT‐1 cells to assess the necessity of the PARN–PTBP1 interaction for maintaining RNA stability. IF staining verified PTBP1 expression in NIT‐1 cells (Figure 6R). The successful knockdown of *Ptbp1* with siRNAs, notably with siRNA2, was confirmed (Figure 6S,T). qRT‐PCR analysis revealed aberrant *Slc30a8* and *Chst3* mRNA levels in NIT‐1 cells lacking *Parn* or *Ptbp1*, which was consistent with findings from β cells with a specific *Parn* deletion (Figure 6U). These results underscored the collaborative role of PARN and PTBP1 in regulating the stability of the *Slc30a8* and *Chst3* mRNAs.

## 3 Discussion

Elevated insulin secretion in response to glucose represents a critical facet in assessing β‐cell function, with compromised insulin secretion identified as a key factor in diabetes pathogenesis.^[^ ^21^ ^]^ Therefore, we investigated the role of PARN in pancreatic β cells through the specific knockout of *Parn* in these cells in mice. Our data indicated that PARN played a vital role in maintaining blood glucose homeostasis in mice by modulating insulin secretion under glucose‐stimulated physiological conditions following *Parn* depletion in β cells. Consequently, we propose that PARN is a pivotal factor involved in sustaining β‐cell function.

The normal development and function of β cells are pivotal determinants of insulin secretion.^[^ ^22^ ^]^ Studies have implicated the RNA‐binding protein fox‐1 homolog in mediating alternative splicing to modulate insulin secretion.^[^ ^23^ ^]^ In our investigation, we observed that PARN loss did not impede β‐cell development but significantly inhibited GSIS, which was attributed to concurrent reductions in insulin maturation and secretion. This assertion is supported by both in vitro and in vivo data.

RBPs pervasively regulate transcripts throughout their life cycle.^[^ ^24^ ^]^ Our results indicated that reduced mRNA stability contributed the diminished levels of gene transcripts associated with insulin synthesis and secretion upon *Parn* deletion. RNA‐seq data revealed significant upregulation and downregulation of a multitude of genes related to insulin secretion, such as *Vsnl1*,^[^ ^25^ ^]^ *Hmgcr*,^[^ ^26^ ^]^ *Hmgn3*,^[^ ^27^ ^]^ and *Nnat*,^[^ ^28^ ^]^ in cKO mice compared with control mice.

Moreover, our LACE‐seq analysis revealed direct binding of PARN to *Slc30a8* and *Chst3*. *Slc30a8* encodes zinc transporter 8 (ZnT8), which is expressed predominantly on the secretory granule membrane of pancreatic β cells, where it governs the synthesis, storage, and secretion of insulin.^[^ ^29^ ^]^ Loss of ZnT8 in mouse models is associated with abnormal dense core formation within insulin granules, leading to impaired GSIS.^[^ ^30^ ^]^ Human studies have linked the *Slc30a8* polymorphism, particularly the rs13266634C allele, to a significant reduction in insulin secretion.^[^ ^31^ ^]^ Notably, conflicting evidence exists, as certain investigations indicate that the loss of *Slc30a8* function may increase insulin secretion.^[^ ^32^ ^]^ A recent study has shown that the diminished activity of ZnT8 in β cells exacerbates human islet amyloid polypeptide (hiAPP)‐induced toxicity, resulting in impaired β‐cell function and disrupted glucose homeostasis under metabolic stress.^[^ ^33^ ^]^ Our study further revealed the binding of PARN to *Chst3*, suggesting its potential role in regulating insulin secretion. Consistent with this discovery, carriers of the C allele at rs4148941 within *Chst3* were shown to have a 32% reduction in glucagon like peptide 1 (GLP‐1)‐stimulated insulin.^[^ ^34^ ^]^

PTBP1, a member of the heterogeneous ribonucleoprotein family, exhibits dynamic shuttling between the nucleus and cytoplasm and plays pivotal roles in diverse cellular processes.^[^ ^35^ ^]^ Its functions include pre‐mRNA alternative splicing and roles in processes such as mRNA polyadenylation, nuclear pore export, mRNA stability, subcellular localization, replication, and internal‐ribosome‐entry‐site‐mediated translation.^[^ ^36^ ^]^ Our investigation revealed an interaction between PARN and PTBP1, which is crucial for maintaining the stability of the *Slc30a8* and *Chst3* mRNAs. Upon glucose stimulation of β cells, PTBP1 undergoes nucleocytoplasmic translocation, where cytoplasmic PTBP1 binds to and stabilizes mRNAs encoding proteins associated with secretory granules (SGs), consequently increasing translation.^[^ ^11^, ^37^ ^]^ Notably, nuclear PTBP1 levels decrease in stimulated nondiabetic islets but remain unaltered in T2DM islets. Moreover, PTBP1 has emerged as a novel risk gene for T2DM linked to diminished insulin secretion.^[^ ^38^ ^]^ These findings underscore PTBP1 as a crucial posttranscriptional regulator of insulin‐related SG components. As *Ptbp1* deletion suppresses GSIS, a plausible hypothesis is that *Parn* deletion may compromise PTBP1 function, contributing to diminished GSIS.

**Figure 7:**
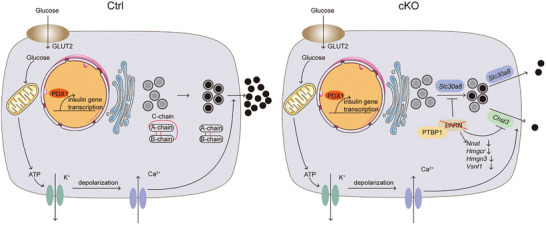
Model of PARN functional mechanisms in mouse islet. PARN and PTBP1 interact to regulate *Slc30a8* and *Chst3* mRNA stability. In PARN‐deficient β cells, reduced *Slc30a8* and *Chst3* mRNA levels lead to fewer mature insulin secretary granules and reduced insulin secretion.

In conclusion, our study elucidated the positive impact of PARN on pancreatic β cells. The diminished RNA stability of *Slc30a8* and *Chst3*, coupled with defects in proinsulin processing and reduced insulin secretion, collectively contributed to impaired GSIS in cKO mice (**Figure** **7**). This insight provides a theoretical foundation for current gene therapy approaches for the treatment of diabetes. However, owing to the substantial RNA requirements of poly(A)‐tail‐primed sequencing (2P‐seq) and the challenges in obtaining islets, this technology was not employed to investigate the 3′ UTR of RNA.

## 4 Experimental Section

### Animals

*Ins2‐Cre* mice were procured from the Jackson Laboratory (JAX:003573), while *Parn* transgenic mice were created via clustered regularly interspaced short palindromic repeats (CRISPR)/CRISPR‐associated protein 9 (Cas9) technology. The single guide RNAs (sgRNAs) were synthesized with a MEGAshortscript T7 Transcription Kit (Ambion) in accordance with the manufacturer's guidelines. For *Parn^f/f^* mice, DNA fragments encompassing exon 7 of the *Parn* gene flanked by two loxP sites and two homology arms served as donor templates. The Cas9 protein (New England Biolabs (NEB), M0646) and sgRNA were coincubated, and the resulting Cas9–sgRNA complex, along with the donor templates, was injected into C57BL/6 zygotes. These manipulated zygotes were then transferred into pseudopregnant CD1 female mice. The resulting founder mice were genotyped and subsequently bred with C57BL/6 mice. HFD‐fed mice were subjected to a 60% high‐fat diet (Hfkbio, 10060, China) from 4 to 18 weeks of age. *Parn* floxed and *Ins2‐Cre* mice were generated via PCR using DNA extracted from their tails. The sgRNA and primer sequences utilized for genotyping are provided in Table S1 (Supporting Information).

All the mice were housed in a controlled environment with standard lighting conditions (12 h light/dark cycle) under the supervision of the Animal Care and Use Committee of China Agricultural University (AW92113202‐3‐3).

### IF and IHC

The pancreas was meticulously isolated and subsequently fixed with a 4% paraformaldehyde solution overnight at 4 °C. Paraffin‐embedded sections were subjected to both IF and IHC analyses. These sections were subjected to antigen retrieval via Tris‐HCl or ethylenediaminetetraacetic acid buffer, followed by a 1 h incubation in 10% normal goat serum at room temperature for blocking. The sections were subsequently incubated overnight at 4 °C with primary antibodies. Gentle washing with phosphate‐buffered saline (PBS) preceded a 1 h incubation with secondary antibodies, both fluorescent and histochemical, at room temperature. IF images were captured using a laser confocal microscope (Nikon, A1), while analysis of the IHC sections was conducted through digital pathology section scanning (Roche, Ventana DP 200). Details of the antibodies utilized are provided in Table S2 (Supporting Information).

### Western Blotting

Tissue lysates were prepared using radio immunoprecipitation assay (RIPA) buffer (Beyotime, P0013B) and subjected to analysis via sodium dodecyl sulfate– polyacrylamide gel electrophoresis (SDS‐PAGE). The proteins were resolved via SDS‐PAGE and subsequently transferred onto polyvinylidene fluoride membranes for 2 h at 4 °C. Next, the membranes were transferred to 5% skim milk, blocked at room temperature for 30 min, and incubated with primary antibodies overnight at 4 °C. After incubation, the membrane was thoroughly washed with Tris‐buffered saline containing Tween and then incubated with secondary antibodies for 1 h at room temperature. To ensure equal loading, the housekeeping protein (glyceraldehyde‐3‐phosphate dehydrogenase, GAPDH) was quantified in each gel. Visualization of the results was accomplished using a chemiluminescent imaging system (Tanon 5200). The specific details of the antibodies used can be found in Table S2 (Supporting Information).

### Intraperitoneal Glucose Tolerance Test (IPGTT), ITT, and GSIS

For the IPGTT, 12 and 16 weeks old mice were fasted for 16 h, followed by intraperitoneal injection of glucose (2 g kg^−1^). Blood glucose levels were assessed in tail blood samples at 0, 20, 40, 60, 80, and 120 min postinjection. In the ITT, mice aged 13 and 17 weeks were subjected to a 4 h fast, after which they received an intraperitoneal injection of insulin (0.75 U kg^−1^). Blood glucose levels were measured in tail blood samples at 0, 20, 40, 60, 80, and 120 min following insulin administration. Serum was collected at fasting and after glucose injection for 10, 20, 30, and 60 min to detect GSIS. Serum insulin levels were quantified using a mouse insulin ELISA kit (Crystal Chem, 90080).

### Isolated Islets

The mice were anesthetized with 1% pentobarbital sodium, and pancreas isolation was achieved by injecting collagenase P (Sigma, 11213857001) into the pancreas through the common bile duct. Following isolation, the digestion process involved shaking at 37 °C and 170 rpm for 8 min, and it was terminated using Hank's buffer (8 g NaCl, 0.4 g KCl, 0.0489 g MgSO_4_, 0.0479 g Na_2_HPO_4_, 0.06 g KH_2_PO_4_, 0.0468 g MgCl_2_, 0.185 g CaCl_2_·2H_2_O, 0.3 g d−(+)‐glucose, 0.35 g NaHCO_3_, and ddH_2_O to 1 L). Islets were subsequently manually selected from the digested tissue. Postisolation, the islets were cultured in Roswell Park Memorial Institute (RPMI) 1640 supplemented with 10% fetal bovine serum (FBS) (VITECH, SE200‐ES) and a penicillin–streptomycin solution (Beyotime, C0222). Groups of ten islets were transferred to cell culture dishes containing 3 or 20 mm glucose Krebs‐Ringer bicarbonate HEPES buffer (KRBH) buffer for 1 h. The intracellular insulin content was determined via acid–alcohol, and the supernatant and intracellular insulin levels were detected by ELISA. Insulin and proinsulin expression levels were analyzed in islets isolated from Ctrl and cKO mice by Western blotting.

### Measurement of β‐Cell Mass and Islet Size

The pancreas was weighed and subsequently fixed. 5 µm sections were obtained at 200 µm intervals for IHC. The β‐cell fractional area was determined by quantifying the percentage of the insulin‐positive pancreatic area in relation to the total pancreatic area for each section. To calculate the β‐cell mass, the β‐cell fractional area was multiplied by the pancreatic weight. The islet distribution ratio was computed as the ratio of the number of islets in different regions to the total number of islets.

### TEM

Isolated mouse islets were initially fixed overnight with 3% glutaraldehyde. The islets were subsequently rinsed with PBS. A second fixation was subsequently performed using 1% osmic acid, followed by additional rinsing with PBS. The islets then underwent a gradient dehydration process involving 50% ethanol, 70% ethanol, 90% ethanol, 90% acetone, and 100% acetone. Sequential soaking in acetone/embedding agent solutions with varying ratios (1:1, 1:2) and acetone/embedding agent ratios (1:3) was subsequently performed. Finally, the tissue was immersed in pure embedding agent for embedding. The resulting embedded blocks were extracted, and ultrathin sections were generated. These sections were subjected to staining with uranyl acetate for 30 min, followed by three rinses with double‐distilled water. Subsequent staining with lead citrate for 10 min was carried out, followed by an additional three rinses with distilled water. Following drying, the sections were observed via transmission electron microscopy (Hitachi, HT7800).

### Cell Culture

The NIT‐1 mouse pancreatic β‐cell line was cultured in RPMI 1640 medium (Gibco, C11875500BT) supplemented with 10% FBS (VITECH, SE200‐ES) and a penicillin–streptomycin solution (Beyotime, C0222). β‐TC6 cells were cultured in high‐glucose Dulbecco's modified Eagle's medium (DMEM; Gibco, C11995500BT). To detect insulin secretion, the cells were pretreated with KRBH containing 3 mm glucose for 1 h, which was then replaced with KRBH containing 20 mm glucose for 1 h. Additionally, the human embryonic kidney cell line HEK293T was cultured in DMEM (Gibco, C11995500BT).

### RNA Sequencing and qRT‐PCR

The total RNA was extracted from isolated islets via the TRIzol method (TaKaRa, RNAiso plus 9109). The RNA integrity and quantity were subsequently and meticulously assessed using an Agilent 2100 bioanalyzer. For sequencing, the fluorescence signal captured by the sequencer was converted into a sequencing peak through computer software, facilitating the acquisition of sequence information for the targeted fragments. Differential expression analysis of the two combinations of DEGs was conducted using DESeq2 software (version 1.20.0). Complementary DNA (cDNA) synthesis was accomplished using a RNA Mini Kit (Tiangen, KR107‐01). qRT‐PCR analysis was performed on a real‐time PCR system (Light Cycle 96 Instrument) with SYBR Green Master Mix (Yeasen, 11198ES03). Detailed information about the primers used can be found in Table S1 (Supporting Information).

### LACE‐Seq

LACE‐seq was executed with islet samples obtained from 12 weeks old mice following a previously outlined protocol.^[^ ^20^ ^]^ In brief, each sample was subjected to UV‐C light irradiation on ice and was administered 3 times for 1 min each to induce cross‐linking. Subsequent to cross‐linking, RNA immunoprecipitation was conducted, and the immunoprecipitated RNA underwent fragmentation and dephosphorylation. The subsequent steps included reverse transcription, strand affinity bead capture of the first‐strand cDNA, poly(A) tailing, pre‐PCR, in vitro transcription, RNA purification, reverse transcription polymerase chain reaction, and deep sequencing.

Upon the acquisition of raw data, an initial filtering process was applied to obtain high‐quality clean data. The clean data were subsequently aligned with the reference genome of the project species (mm10), and whole‐genome peak calling was subsequently executed on the resulting alignment results. The binding preferences of the proteins for RNA were investigated, and the binding sites were subjected to motif analysis.

### Plasmid Construction and Transfection

For amplification of exogenous DNA, PCR was employed, followed by vector cleavage via restriction enzymes. The resulting sequences were subsequently joined through homologous recombination using the Seamless Cloning Kit (Beyotime, D7010M). Prior to transfection, the cell density reached ≈70–80%, and fresh culture medium (2 mL) was added to each well of a six‐well plate. Subsequently, antibiotic‐free (125 µL) and serum‐free medium, along with plasmid DNA (2.5 µg), were combined in a centrifuge tube supplemented with Lipo8000 (Beyotime, C0533) (4 µL) transfection reagent, thoroughly mixed, and uniformly introduced into the wells.

The synthesis of siRNAs was outsourced to GenePharma, with careful attention given to RNA enzyme contamination during transfection. At a cell density of ≈60% in a 12‐well plate, the culture medium was replaced with fresh medium (800 µL). In a separate RNA‐free centrifuge tube, culture medium (100 µL) without antibiotics or serum and GP‐transfect‐Mate (GenePharma, G04008) (5 µL) were combined. Simultaneously, basic culture medium (100 µL) and siRNA (80 pmol) were mixed in another RNA‐free centrifuge tube. After thorough mixing and a 15 min incubation at room temperature, the resulting mixture was uniformly introduced into the medium for a 48 h incubation, after which the knockdown effect was assessed. The primers and siRNA sequences utilized are detailed in Table S1 (Supporting Information).

### ActD Treatment

NIT‐1 cells were transfected with *Parn* siRNA for 24 h and then incubated with 4 µg mL^−1^ ActD (Sigma, SBR00013). Samples were collected at various time points to measure the mRNA expression levels of *Slc30a8* and *Chst3* via qRT‐PCR.

### Co‐IP

Islet samples from 12 weeks old mice served as the source for immunoprecipitation (IP) mass spectrometry samples. Total protein extraction was conducted using RIPA buffer (Beyotime, P0013). The magnetic beads were incubated with an anti‐PARN antibody or immunoglobulin G (IgG) at room temperature for 1 h, followed by washing and overnight incubation with the protein sample at 4 °C. Protein identification was carried out using a Thermo‐Q Exactive high‐resolution mass spectrometer, and the raw data were preprocessed via Mascot Distiller 2.4.

For protein extraction from cotransfected cells, RIPA lysis buffer was used. The extracts were incubated with anti‐FLAG magnetic beads (Beyotime, P2102) at 4 °C for 6 h. The proteins were subsequently subjected to IP with a monoclonal anti‐FLAG antibody or anti‐HA antibody and subjected to analysis via Western blotting. Detailed information regarding the antibodies utilized can be found in Table S2 (Supporting Information).

### Dual‐Fluorescence Reporting System

The plasmids psiCheck2‐*SLC30A8* WT (Mut) and psiCheck2‐*Chst3* WT (Mut) were constructed with the BGI gene. The media was removed from the transfected HEK293T cells cultured in 12‐well plates, each well was washed once with 1 mL PBS, and the PBS was removed. Next, 150 µL of 1 × Passive Lysis Buffer was added to each cell culture well, and the lysed cells were shaken slowly at room temperature for 15 min. Then, 10 mL of Luciferase Assay Buffer II was added to the Luciferase Assay Substrate and mixed well. A volume of 100 µL of the mixture was added to a 96‐well fluorescence detection plate. A total of 20 µL of cell lysate was collected from each cell culture well into the detection well and mixed well to detect firefly luciferase activity. Renilla luciferase activity was measured by adding 200 µL of Stop & Glo substrate to 10 mL of Stop & Glo buffer, mixing the substrate, and adding 100 µL of reaction mixture to the wells of the assay. The activity signals of Renilla luciferase and firefly luciferase were detected and collected according to the instructions supplied with the microplate multiplate reader (Tuner Biosystems, 9300‐062).

### Statistical Analysis

Statistical analyses were conducted using Prism 9.0 software. For unpaired comparisons between two groups, the two‐tailed Student's *t*‐test was employed to calculate the respective *p* values. In cases involving three or more groups, comparisons were carried out via two‐way analysis of variance (ANOVA) followed by the Tukey multiple comparisons test, facilitating evaluations among all pairs of columns. The results were presented as the means ± standard deviations. The sample size estimation was derived from the anticipated effect size on the basis of prior experiments, as specified in the legend. No blinding method was employed during the study. Significance levels were denoted as follows: *p < 0.05, **p < 0.01, ***p < 0.001, and ****p < 0.0001.

## Conflict of Interest

The authors declare no conflict of interest.

## Author Contributions

X.X., J.C., and J.L. conceived and designed the entire project. X.X., X.C., C.W., L.S., Z.L., and S.T. performed the experiments. X.X., X.C., S.T., and F.W. generated and bred the knockout mice. X.X., L.S., W.Y., X.Y., J.C., and J.L. analyzed the data. X.X., F.W., D.D., J.C., and J.L. wrote the paper. All authors read and approved the final paper.

## Supporting information

Supporting Information

## Data Availability

The data underlying this article are available in GEO dataset, at https://www.ncbi.nlm.nih.gov/geo/query/acc.cgi?&acc=GSE248389 and https://www.ncbi.nlm.nih.gov/geo/query/acc.cgi?acc=GSE248559. A link to a UCSC genome browser session displaying the uploaded sequence tracks (https://genome.ucsc.edu/s/xxm/mm100).
