# Supplementary material for: PARN Maintains RNA Stability to Regulate Insulin Maturation and GSIS in Pancreatic β Cells
Source: Adv Sci (Weinh). 2024 Sep 19;11(42):2407774. doi: 10.1002/advs.202407774 (PMC11558150; doi:10.1002/advs.202407774)
Supplement: Supplementary file 1 — Supporting Information [file ADVS-11-2407774-s001.docx]

**Supplementary table1.**

| Primer | Sequence |
| --- | --- |
| *Ptbp1*-plasmid-F | ATGGACGGCATCGTCCCAGA |
| *Ptbp1*-plasmid-R | CTAGATGGTGGACTTGGAAA |
| *Parn*-JD-F | CACTTGAGAGGCAGTGAATTTGAGGC |
| *Parn*-JD-R | TGGATCTGGAG-TTCTGGGCATCTG |
| *Ins2*-JD-10022 | ACTCCAAGTGGAGGCTGAGA |
| *Ins2*-JD-10023 | TCCTTCCACAAACCCATAGC |
| *Ins2*-JD-7338 | CTAGGCCACAGAATTGAAAGATCT |
| *Ins2*-JD-7339 | GTAGGTGGAAATTCTAGCATCATCC |
| *Parn*-F | AGTGTCCTGTGCTGTTTCGT |
| *Parn*-R | CATTCAGCTCCTCCTGCTCC |
| *Vsnl1*-F | CAGGGCGCAATCCAAGAGAG |
| *Vsnl1*-R | CCCACCATTTTGTAGATAGCCTCG |
| *Nnat*-F | TGCGAGAAGTGAGGTGTTCAG |
| *Nnat*-R | GGTGCCTACGCCCATATCTC |
| *Hmgcr*-F | ACGATCCTTCCTTATTGGCGG |
| *Hmgcr*-R | AAGAGGCCAGCAATACCCAG |
| *Hmgn3*-F | TGTTCCACCAAAACCGGAGT |
| *Hmgn3*-R | CAGTTCTCTGTGCCTTCCTCT |
| *Cpeb1*-F | GAACAACAGCTTCGCTCACC |
| *Cpeb1*-R | TCCTGCTGCTTCTTCCTGC |
| *Sytl4*-F | CCTGGGTTGCTGGTCTAAGT |
| *Sytl4*-R | AAGAGTGACCAGATGTGTCTGT |
| *Ptbp1*-F | TAGCAGTCGGTACAAAGCGG |
| *Ptbp1*-R | CGTTTCCATTGGCTGCTGAG |
| *Slc30a8*-F | GGACAGCGCATCAAACATCA |
| *Slc30a8*-R | GCTTCTGTCGAAGTTCTCTGTC |
| *Chst3*-F | GTGGAGTGAGGTGGGGAGTC |
| *Chst3*-R | GCTTGTCGGAGACCCTGGA |
| *Parn*-mus-387-F (siRNA1) | GCAUGUAACGAAGUCAUUUTT |
| *Parn*-mus-387-R (siRNA1) | AAAUGACUUCGUUACAUGCTT |
| *Parn*-mus-776-F (siRNA2) | GGAAGUAUCCUAAAGGCAUTT |
| *Parn*-mus-776-R (siRNA2) | AUGCCUUUAGGAUACUUCCTT |
| *Parn*-mus-1398*-*F (siRNA3) | GGUCAUGGAUAUUCCCUAUTT |
| *Parn*-mus-1398*-*R (siRNA3) | AUAGGGAAUAUCCAUGACCTT |
| *Parn*-mus-1443*-*F (siRNA4) | GCCUAAGCGGGACCAUGUUTT |
| *Parn*-mus-1443*-*R (siRNA4) | AACAUGGUCCCGCUUAGGCTT |
| *Ptbp1*-mus-391-F (siRNA1) | GCAGCCAAUGGAAACGAUATT |
| *Ptbp1*-mus-391-R (siRNA1) | UAUCGUUUCCAUUGGCUGCTT |
| *Ptbp1*-mus-595-F (siRNA2) | GCUGCCAACACUAUGGUUATT |
| *Ptbp1*-mus-595-R (siRNA2) | UAACCAUAGUGUUGGCAGCTT |
| *Ptbp1*-mus-902-F (siRNA3) | GCACCGUCCUGAAGAUCAUTT |
| *Ptbp1*-mus-902-R (siRNA3) | AUGAUCUUCAGGACGGUGCTT |
| *Ptbp1*-mus-1656-F (siRNA4) | GCUGCACCGCUUCAAGAAATT |
| *Ptbp1*-mus-1656-R (siRNA4) | UUUCUUGAAGCGGUGCAGCTT |
| *Slc30a8*-mus-772-F (siRNA1) | GGCUGACAUUUGGGUGGUATT |
| *Slc30a8*-mus-772-R (siRNA1) | UACCACCCAAAUGUCAGCCTT |
| *Slc30a8*-mus-889-F (siRNA2) | GCAGCCAACAUUGUACUAATT |
| *Slc30a8*-mus-889-R (siRNA2) | UUAGUACAAUGUUGGCUGCTT |
| *Slc30a8*-mus-998-F (siRNA3) | GGGAUGUAUUUCAGAGCAUTT |
| *Slc30a8*-mus-998-R (siRNA3) | AUGCUCUGAAAUACAUCCCTT |
| *Slc30a8*-mus-1111-F (siRNA4) | GCCAGCACCGUCAUGAUCUTT |
| *Slc30a8*-mus-1111-R (siRNA4) | AGAUCAUGACGGUGCUGGCTT |
| *Chst3*-mus-716-F (siRNA1) | GACCUUGUACACAGCCUAATT |
| *Chst3*-mus-716-R (siRNA1) | UUAGGCUGUGUACAAGGUCTT |
| *Chst3*-mus-1533-F (siRNA2) | GGUUGGAUCUACGAGUCAUTT |
| *Chst3*-mus-1533-R (siRNA2) | AUGACUCGUAGAUCCAACCTT |
| *Chst3*-mus-1583-F (siRNA3) | GCUUCACGCAUAGUGGCCUTT |
| *Chst3*-mus-1583-R (siRNA3) | AGGCCACUAUGCGUGAAGCTT |
| *Chst3*-mus-1884-F (siRNA4) | GCGACAGCAGCGAUGUCUATT |
| *Chst3*-mus-1884-R (siRNA4) | UAGACAUCGCUGCUGUCGCTT |
| gRNA-*Parn ^f/f^* | cgcacagctcagacgttgagagg |

**Supplementary table 2.**

| Antibody | Company | Cat. |
| --- | --- | --- |
| PARN | Proteintech | 13799-1-AP |
| Insulin | Proteintech | KHC0004 |
| Proinsulin | DSHB | GS-9A8 |
| Glucagon | Boster | BM1621 |
| PARN | Abcam | ab125185 |
| FLAG | Proteintech | 66008-4-Ig |
| HA | Abmart, | 26D11 |
| GAPDH | Proteintech | 60004-1-Ig |
| PTBP1 | Santa Cruz | sc-56701 |
| SLC30A8 | Proteintech | 16169-1-AP |
| CHST3 | Proteintech | 18242-1-AP |
| Rabbit IgG | Beyotime | A7058 |


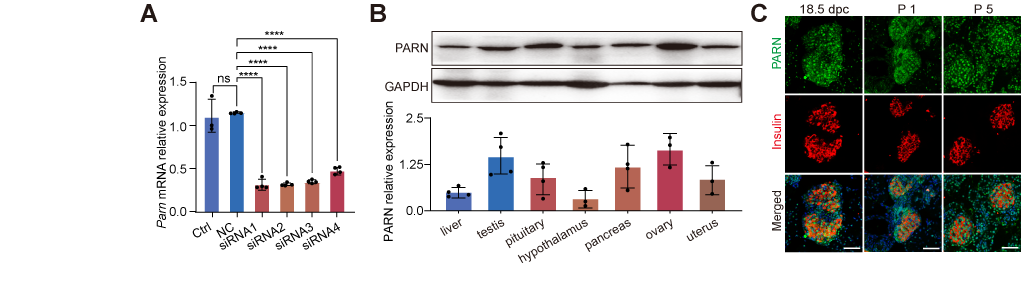


**SFig.1 PARN was highly expressed in β cells**. (A) qRT-PCR verified the knockdown of *Parn* mRNA in NIT-cells 48 hours after siRNA transfection, n=3-4. (B) Western blotting was detected PARN expression of mouse tissue. Bars represent the average protein expression levels normalized to GAPDH obtained by densitometry analysis, n=3-4. (C) The expression pattern of PARN in mouse pancreas at different periods was detected by immunofluorescence with antibody against Insulin (red), PARN (green), the nuclei were stained DAPI (blue), bar=25 μm. **** *P*<0.0001, Student’s *t*-test and ANOVA.


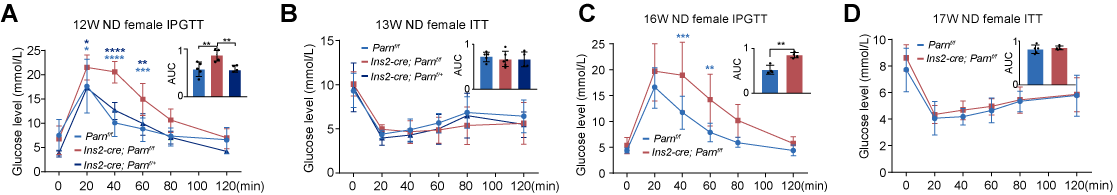


**SFig.2 β-cell specific deletion of PARN caused glucose intolerance in female ND mice.** (A) The IPGTT test (2 g glucose/kg body weight) was carried out in 16-h fasted 12-wk-old female mice, area under the curve (AUC), n=4-5. (B) The ITT test (0.75 U insulin/kg body weight) was carried out in 4-h fasted 13-wk-old female mice, area under the curve (AUC), n=3-5. (C) Variations in glucose levels during IPGTT at 16 weeks, area under the curve (AUC), n=4-5. (D) ITT at 17 weeks. The insulin dose was 0.75 U/g body weight, area under the curve (AUC), n=5. * *P*<0.05, ** *P*<0.01, *** *P*<0.001, **** *P*<0.0001, Student’s *t*-test and ANOVA.


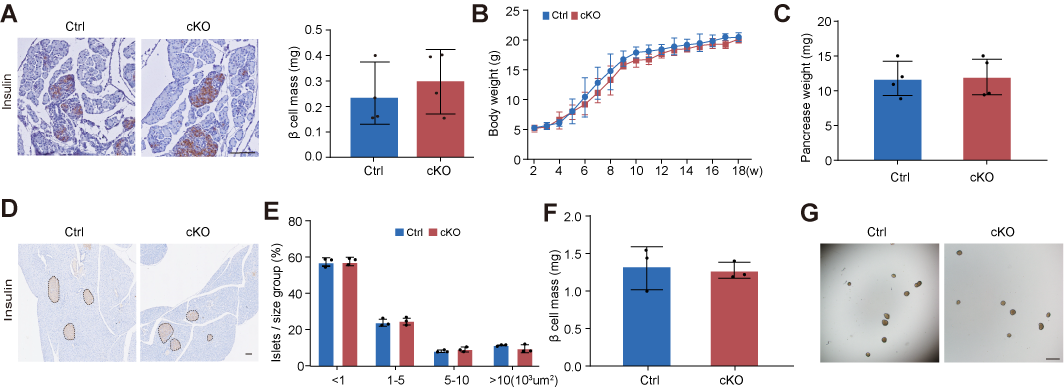
 **SFig.3 β-cell-deletion PARN did not affect cell development in female mice under normal diet conditions.** (A) Immunohistochemistry pancreatic sections from 3-days mice. Results of morphometric analysis of β cell mass at 3-days mice, n=3-4, bar=100 μm. (B) Body weight from 2 weeks to 18 weeks of Ctrl and cKO mice, n=4. (C) Pancreas weight at 18 weeks of Ctrl and cKO mice, n=4. (D) Immunohistochemistry pancreatic sections from 18-week mice, bar=100 μm. (E) β cell mass in entire pancreas in 18 weeks mice, n=3. (F) Islet size distribution as stratified by ranges of islet area from 18 weeks mice by image J, n=3. (G) Islets were isolated from 18-week-old Ctrl and cKO male mice. Student’s *t*-test and ANOVA.


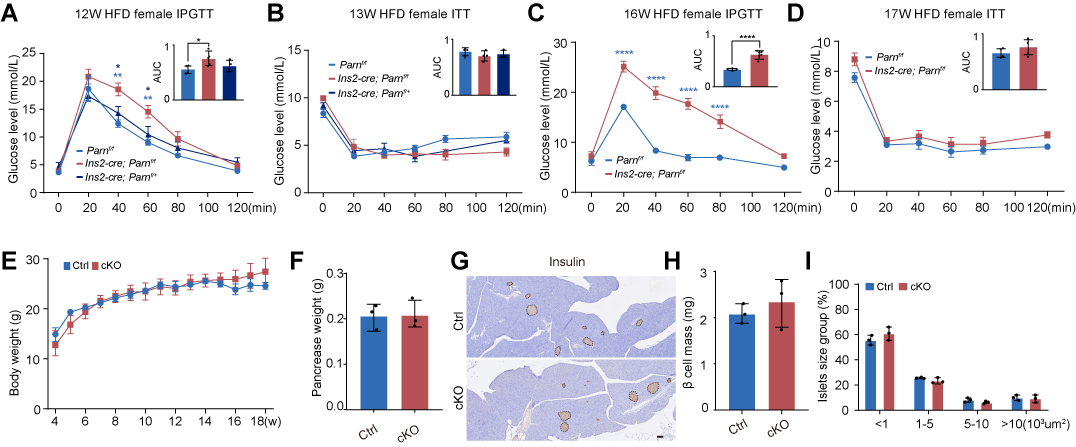


**SFig.4 β-cell specific knockout *Parn* induces glucose intolerance in high fat diet female mice.** (A) IPGTT, 2 g/kg glucose in 16 h fasted 12 weeks mice and the area under the curve (AUC), n=4-5. (B) ITT, 0.75 U/kg insulin in 4 h fasted 13 weeks mice and the area under the curve (AUC), n=3-4. (C) IPGTT, 2 g/kg glucose in 16 h fasted 16 weeks mice and the area under the curve (AUC), n=4-5. (D) ITT, 0.75 U/kg insulin in 4 h fasted 17 weeks mice, the area under the curve (AUC) of ITT performed at 17 weeks, n=4. (E) Body weight from 4 weeks to 18 weeks of Ctrl and cKO mice, n=4-6. (F) Pancreas weight from 18 weeks Ctrl and cKO HFD female mice, n=3. (G)Immunohistochemistry pancreatic sections from 18-week Ctrl and cKO mice., bar=100 μm. (H) β cell mass in entire pancreas at 18 weeks Ctrl and cKO mice, n=3. (I) Islet size distribution as stratified by ranges of islet area at 18 weeks mice by ImageJ, n=3. * *P*<0.05, ** *P*<0.01, **** *P*<0.0001, Student’s *t*-test and ANOVA.


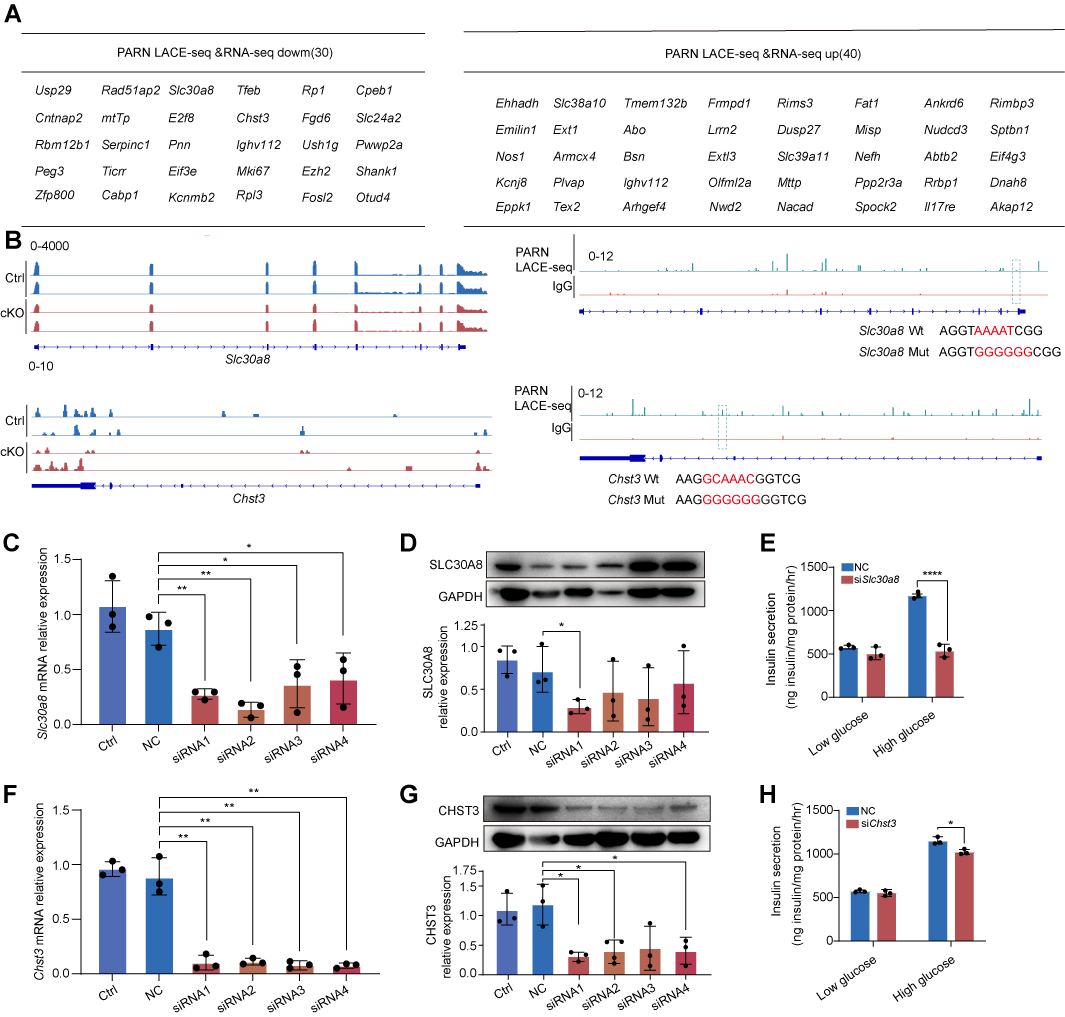


**SFig.5 PARN regulates mRNA expression in islets.** (A) LACE-seq and RNA-seq overlap gene. (B) IGV visualizes gene abundance in Ctrl and cKO and location of PARN binding peaks and base mutation sequence. (C) The *Slc30a8* mRNA expression level of NIT-1 cells transfected with *Slc30a8* siRNA was detected by qRT-PCR, n=3. (D) The knockdown efficiency of NIT-1 cells transfected with *Slc30a8* siRNA was detected by Western blotting, n=3. (E) The insulin secretion level of NIT-1 cells transfected with *Slc30a8* siRNA1 was detected by ELISA kit, n=3. (F) The knockdown efficiency of NIT-1 cells transfected with *Chst3* siRNA was detected by qRT-PCR, n=3. (G) Western blotting analysis of protein expression in NIT-1 cells transfected with *Chst3* siRNA, n=3-4. (H) ELISA kit was used to detect the insulin level secretion of NIT-1 cell transfected *Chst3* siRNA1, n=3. * *P*<0.05, ** *P*<0.01, **** *P*<0.0001, Student’s *t*-test and ANOVA.
